# Nanoflower-shaped cobalt-metal–organic framework as an oxidase-like nanozyme and 1,2-diaminobenzene as a catalytic substrate for the innovative signal “off-on” aptamer sensing of prostate-specific antigen

**DOI:** 10.1039/d6ra01552b

**Published:** 2026-05-05

**Authors:** Chen Ji, Yi Zhang, Xingtian Wang, Pingying Xie, Shaoting Wu, Yanfang Zheng, Mingqing Huang

**Affiliations:** a Department of Urology, The Affiliated People's Hospital of Fujian University of Traditional Chinese Medicine Fuzhou 350004 China; b The Affiliated People's Hospital, College of Pharmacy, Fujian University of Traditional Chinese Medicine Fuzhou 350122 China hmq1115@126.com yfzheng@fjtcm.edu.cn; c Department of Clinical Neurophysiology, The Affiliated People's Hospital of Fujian University of Traditional Chinese Medicine Fuzhou 350004 China 815181526@qq.com

## Abstract

The development of simple, sensitive, and reliable methods for detecting prostate-specific antigen (PSA) holds significant importance for the early screening and diagnosis of prostate cancer. In this work, a novel signal “off-on” electrochemical aptamer (Apt) sensing platform was constructed for the first time to detect PSA, utilizing a synthesized nanoflower-shaped three-dimensional cobalt-metal–organic framework (Co-MOF) as an oxidase-mimicking nanozyme and employing 1,2-diaminobenzene as the catalytic substrate. In this design, the Co-MOF nanozyme can directly catalyze the oxidation of 1,2-diaminobenzene to generate diaminophenazine (DAP), an electroactive substance, without the need for H_2_O_2_. However, when the prepared Co-MOF nanozyme binds to PSA-specific aptamers, its enzyme-like activity becomes inhibited due to the blockage of active sites by the aptamer, leading to the disappearance of the DAP current that corresponds to the signal “off” state. Notably, in the presence of PSA, the catalytic activity of the Co-MOF is restored as the specific binding between Apt and PSA causes Apt to detach from the Co-MOF surface, resulting in the recovery of the DAP current and switching the signal to the “on” state. After the optimization of key experimental parameters, the proposed “off-on” nanozyme-based electrochemical aptasensor demonstrates excellent PSA detection performance. Moreover, this platform may provide a novel, simple, and reliable strategy for detecting a wide range of biomarkers by simply replacing the corresponding aptamer.

## Abstract 2

In this work, an innovative “off-on” sensing strategy controlled by Apt was developed for the detection of PSA based on a Co-MOF nanohybrid, and the proposed sensor exhibited excellent detection performance.

## Graphical abstract

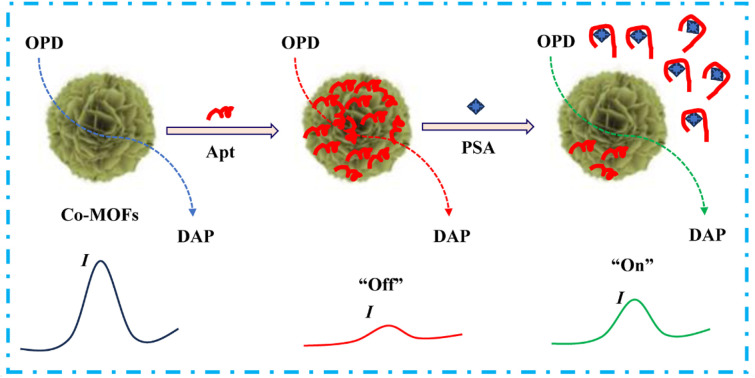

## 1. Introduction

Prostate-specific antigen (PSA) serves as a key cancer biomarker for the early diagnosis and subsequent management of prostate cancer. Its sensitive and efficient determination is crucial for early clinical screening, disease prevention, and the monitoring of pathological progression. Generally, the normal concentration of PSA in a healthy person is below 4 ng mL^−1^.^1–3^ Consequently, a variety of analytical techniques with high selectivity and sensitivity have been developed, including luminescence immunoassays, enzyme-linked immunosorbent assays, surface plasmon resonance analysis, fluorescent immunoassays, and surface-enhanced Raman scattering.^4–6^ Nevertheless, these approaches often demand specialized operators and costly instrumentation, in addition to being complex and time-intensive.^7^ As a result, electrochemical and colorimetric methods, known for their simplicity, speed, and cost-effectiveness, are attracting increasing attention. Indeed, several innovative electrochemical sensing platforms have recently been designed for PSA detection,^8–10^ yet they involve elaborate procedures for signal labeling and the immobilization of recognition elements such as antibodies, DNA, and Apt.

In recent years, the emergence of nanozymes has established a dynamic research frontier in electrochemical sensing.^11,12^ Unlike natural enzymes, artificial nanozymes provide distinct advantages such as easy availability, robust stability, and low cost, thereby endowing nanozyme-based electrochemical sensors with promising potential for applications in food safety, environmental surveillance, and disease diagnostics.^13–15^ More recently, aptamer-regulated “off-on” sensing strategies employing peroxidase (POD)-mimicking nanozymes have attracted considerable interest as they eliminate the need for complex labeling and receptor immobilization procedures.^16–19^ Nevertheless, such POD-like nanozyme systems still rely on the presence of hydrogen peroxide (H_2_O_2_) during catalysis, which introduces safety concerns and limits their practical use.^20,21^ In contrast, oxidase (OXD)-mimicking nanozymes are capable of catalyzing substrate oxidation using ambient oxygen rather than H_2_O_2_, offering a safer and more convenient alternative for biosensing, anti-inflammatory, and antibacterial applications.^22,23^ Consequently, advancing “off-on” sensing platforms based on OXD-like nanozymes holds significant value for the design of innovative sensor systems.

**Scheme 1:**
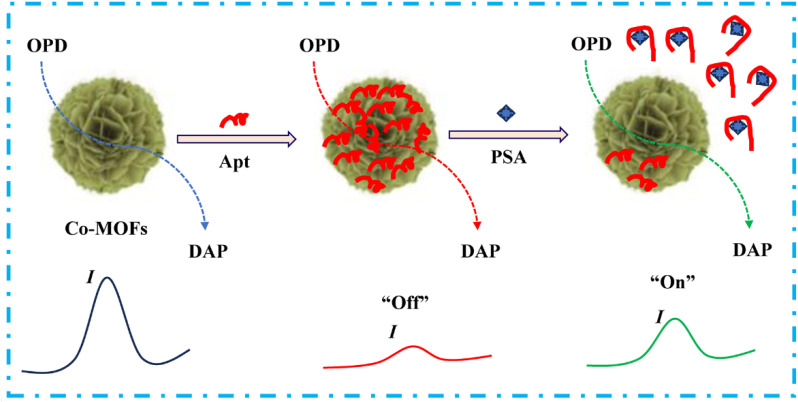
Schematic of the signal “off-on” electrochemical Apt sensing of PSA based on the Co-MOF nanozyme.

In the present work, a nanoflower-shaped three-dimensional cobalt–metal–organic framework (Co-MOF) was synthesized for the first time *via* a simple solvothermal approach. Characterization results demonstrated that the obtained Co-MOF nanozyme exhibits distinct oxidase-like catalytic activity, enabling the oxidation of 1,2-diaminobenzene (OPD) to produce diaminophenazine (DAP) without the need for H_2_O_2_. Upon immobilizing the PSA-specific Apt onto the surface of the Co-MOF, the OXD-like activity was effectively suppressed due to the blockage of active sites, leading to the disappearance of the DAP oxidation peak—corresponding to the signal “off” state. Notably, the catalytic activity could be restored following the introduction of PSA as the specific binding between the Apt and PSA causes Apt to detach, thereby recovering the DAP current and switching the signal to the “on” state. Leveraging this mechanism, a novel “off-on” electrochemical aptasensor based on this nanozyme was developed for the simple and sensitive detection of PSA (Scheme 1). This platform not only circumvents the conventional requirement for aptamer immobilization but also holds considerable promise for the early diagnosis of prostate cancer and the detection of a wide range of biomarkers beyond PSA.

## 2. Experimental section

### 2.1 Reagents and apparatus

PSA was purchased from Sigma-Aldrich (China), while bovine serum albumin (BSA), lysozyme (Lys), glutathione (GSH), and thrombin (TB) were sourced from Sangon Biotech Inc. Co(NO_3_)_2_·6H_2_O, 2-methylimidazole (2-MI), and OPD were obtained from Sinopharm Chemical Reagent Co., Ltd, while methanol (MeOH) was supplied by Shanghai Macklin Biochemical Technology Co., Ltd The PSA-specific Apt was procured from Sangon Biotech Inc (Shanghai, China). All reagents were of analytical grade and used as received. Electrochemical measurements were carried out on a CHI600F electrochemical workstation (China).

### 2.2 Synthesis of the Co-MOF

The Co-MOF was synthesized according to a previously reported procedure.^24,25^ In brief, 1.5 mM Co(NO_3_)_2_·6H_2_O was dissolved in 30 mL of MeOH and magnetically stirred for 5 minutes. Subsequently, 10 mL of an MeOH solution containing 3 mM 2-MI was added dropwise under continuous magnetic stirring for an additional 5 minutes. The resulting mixture was transferred to a 75 mL Teflon-lined autoclave and heated at 120 °C for 12 hours, followed by natural cooling to ambient temperature. Finally, the obtained Co-MOFs was washed sequentially with ethanol and deionized water and dried overnight at 60 °C.

### 2.3 Apt sensor fabrication and PSA detection

A polished glassy carbon electrode (GCE) was first modified with 10 µL of a Co-MOF nanozyme suspension (0.5 mg mL^−1^). The resultant Co-MOF/GCE was then incubated in an Apt solution, allowing Apt to adsorb onto the surface and form Apt/Co-MOF/GCE. Subsequently, Apt/Co-MOF/GCE was incubated with varying concentrations of PSA solutions (50 µL), and the final electrode was tested in an oxygen-saturated acetate buffer (0.1 M) containing OPD using differential pulse voltammetry (DPV).

## 3. Results and discussion

### 3.1 Characterization of the Co-MOF

**Fig. 1:**
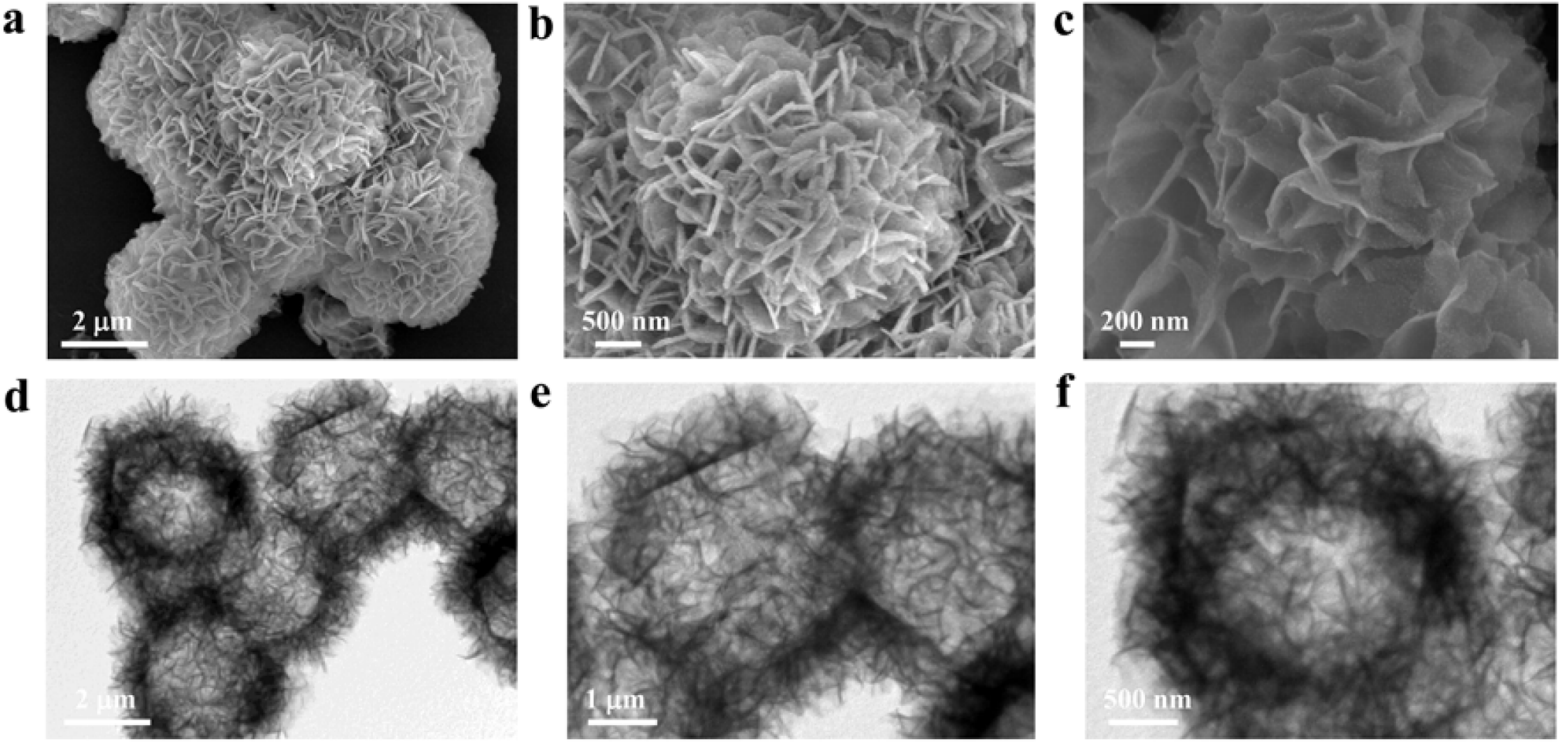
SEM (a–c) and TEM (d–f) images of the Co-MOF.

The Co-MOF nanohybrid was synthesized *via* the hydrothermal treatment of a mixture containing 2-MI and Co(NO_3_)_2_·6H_2_O, as depicted in Scheme S1. The morphology and structure of the resulting materials were characterized by SEM and TEM (Fig. 1). The SEM images clearly show that the as-synthesized Co-MOF possesses a three-dimensional nanoflower-like architecture assembled from two-dimensional nanosheets, with an average diameter of approximately 3 µm, which is consistent with the TEM observations. Energy-dispersive X-ray spectroscopy (EDS) elemental mapping further confirmed the uniform distribution of Co, N, O, and C throughout the material (Fig. S1). The crystalline phase of the Co-MOF was examined by powder X-ray diffraction (PXRD), which showed a characteristic peak at about 10.42° (Fig. S2), corresponding to the typical diffraction pattern of this material. Fourier-transform infrared (FTIR) spectroscopy was used to investigate the molecular structure and chemical composition. As shown in Fig. S3, peaks at 2823 and 2926 cm^−1^ correspond to the C–H stretching vibrations, while the band at 3465 cm^−1^ is associated with the surface hydroxyl (–OH) groups. Additionally, peaks at 1350 and 1632 cm^−1^ are attributed to the Co–O bond. These findings align well with the previously reported data,^23,24^ confirming the successful synthesis of the Co-MOF. In addition, the fabricated Co-MOF/GCE was characterized by electron microscopy, and Fig. S4 shows that a considerable amount of Co-MOF nanohybrids is dispersed on the surface of GCE, confirming the successful immobilization of the nanozymes onto the GCE surface.

### 3.2 Detection principle and feasibility

The detection principle can be summarized as follows: (a) the Co-MOF nanozyme exhibits significant OXD-type catalytic activity, enabling the oxidation of OPD to generate DAP, which produces a detectable current signal. (b) After immobilizing the PSA-related Apt onto the Co-MOF surface through simple adsorption and electrostatic interactions, the nanozyme activity is suppressed, leading to the disappearance of the DAP peak (signal “off”). (c) Upon introduction of PSA, the catalytic activity of Apt/Co-MOF is restored, and the DAP current increases correspondingly (signal “on”).

**Fig. 2:**
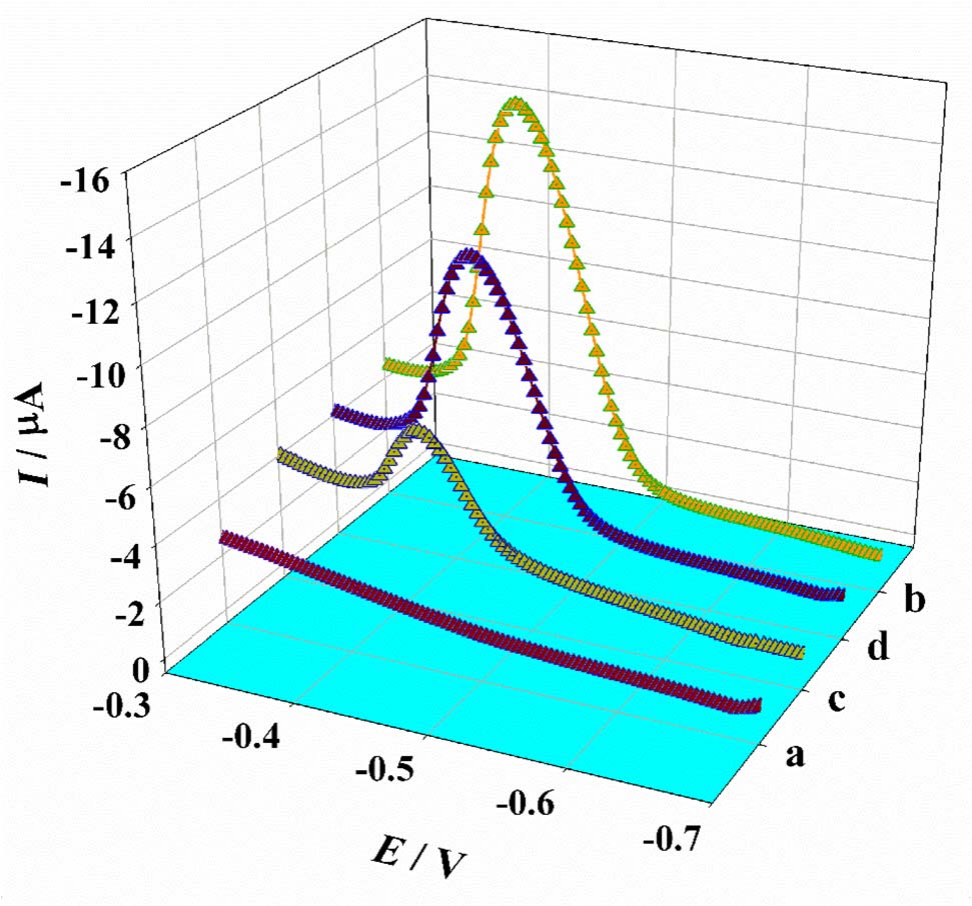
DPV response signals of DAP at bare GCE (a), Co-MOF/GCE (b), and Apt/Co-MOF/GCE before (c) and after (d) incubation with PSA.

Fig. 2 shows the DPV responses of different electrodes in a buffer solution containing OPD with saturated oxygen. No DAP oxidation peak is observed on the bare GCE within the scanned potential range, indicating that the GCE alone does not catalyze OPD oxidation to produce DAP. In contrast, the as-synthesized Co-MOF exhibits a distinct reduction peak at approximately −0.41 V, demonstrating that the Co-MOF nanozyme possesses notable OXD-like activity and can catalyze OPD oxidation in the absence of H_2_O_2_. When Apt is adsorbed onto the Co-MOF surface to form Apt/Co-MOF/GCE, the catalytic activity is inhibited, resulting in the loss of the DAP current (signal “off”). Notably, this activity is recovered after the addition of PSA, and the reduction peak reappears. This restoration is attributed to the specific binding between Apt and PSA, which causes Apt to detach from the nanozyme surface. The sensing principle was characterized by electrochemical impedance spectroscopy. As shown in Fig. S5, the charge transfer resistance (*R*_ct_) is represented by the diameter of the semicircle in the Nyquist plot. Fitting the EIS data to an equivalent circuit revealed that the *R*_ct_ value for Co-MOF/GCE is relatively small, while a marked increase in *R*_ct_ is observed for Apt/Co-MOF/GCE. This rise in resistance can be attributed to the generally low conductivity of Apt. In the presence of PSA, the *R*_ct_ value decreased, thus confirming the successful construction of the Apt/Co-MOF sensor. Based on this behavior, a signal “off-on” electrochemical aptasensor for PSA was developed using the Co-MOF as an OXD-mimicking nanozyme and OPD as the catalytic substrate.

### 3.3 PSA detection

**Fig. 3:**
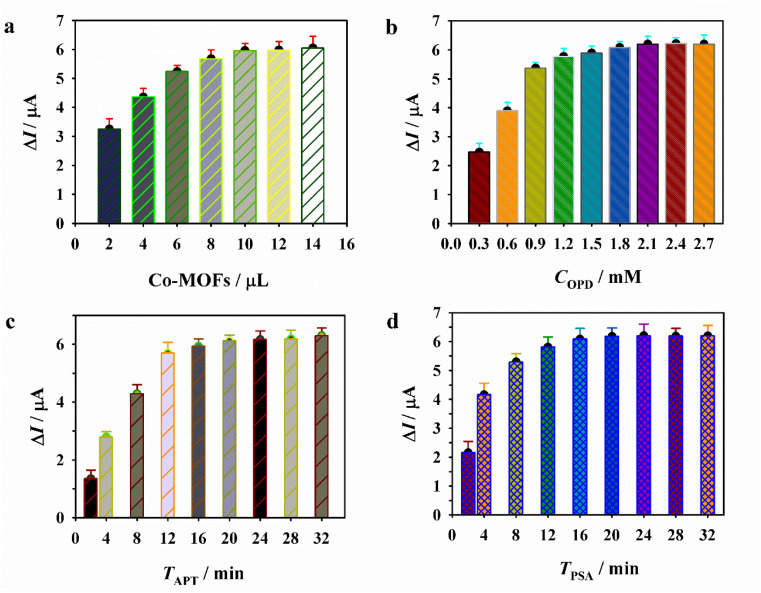
Optimization of the experimental conditions for PSA detection: Effects of the (a) amount of Co-MOF nanozyme, (b) OPD concentration, and incubation time of (c) Apt and (d) PSA.

**Fig. 4:**
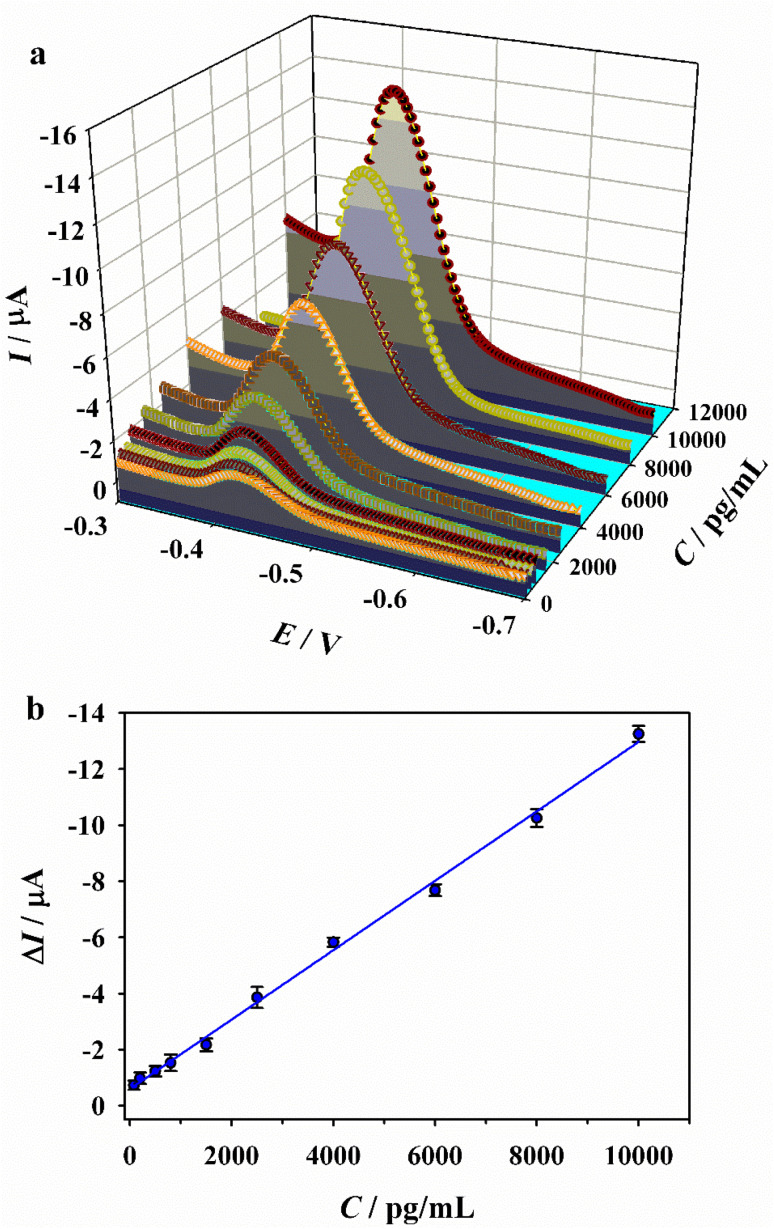
(a) DPV responses of DAP current based on Apt/Co-MOF/GCE towards PSA with different concentrations (b) and the corresponding linear relationships for PSA detection.

Prior to the quantitative detection of PSA, several key experimental conditions were optimized to enhance detection sensitivity, including the amount of the Co-MOF, the concentration of OPD, and the incubation times for Apt and PSA. The difference in DAP current (Δ*I*) before and after the addition of PSA was considered the analytical signal. As shown in Fig. 3, the optimal parameters were determined as follows: the amount of the Co-MOFs, 10 µL; OPD concentration, 1.5 mM; Apt incubation time, 16 min; and PSA incubation time, 20 min. The Apt-controlled signal “off-on” sensor was subsequently employed for the detection of PSA at varying concentrations. As shown in Fig. 4a, the DAP current gradually increased with increasing PSA levels, indicating that PSA can restore the OXD-like catalytic activity of the Co-MOF nanozyme. A linear relationship was observed between the Δ*I* values and the logarithm of PSA concentration over the range of 80 to 10 000 pg mL^−1^. The corresponding regression equation is *y* = −0.5645 − 0.0012*x* (*R*^2^ = 0.9963) (Fig. 4b), with a calculated limit of detection (LOD) of 20 pg mL^−1^ (S/N = 3). Compared with the previously reported electrochemical PSA sensors (Table 1), the platform developed in this study offers a comparable or lower LOD along with a wider linear range. This excellent detection performance can be attributed to the superior OXD-like activity of the Co-MOF nanozyme. Notably, the “off-on” sensing design eliminates the need for complex labeling and receptor immobilization steps, thereby simplifying the overall detection procedure and enhancing operational ease.

**Table 1:** Comparison of the performance of the as-developed Apt/Co-MOF sensor with those of the previously reported electrochemical sensors for PSA

Sensors	Methods	Linearities (pg mL^−1^)	LODs (pg mL^−1^)	Ref.
Ab/HO-BN/COOH-CDs@AuNPs	DPV	1–3.5 × 10^4^	0.136	26
Apt/GA/AuNPs/Nafion	DPV	50–5 × 10^4^	30.6	27
Ab/AuNPs/rGO	Amperometry	0.1–1 × 10^7^	30	28
Ab/AuNRs/rGO	DPV	100–1.5 × 10^4^	16	29
Ab/Cys-AuNP	DPV	5 × 10^3^–2.5 × 10^5^	470	30
Ab/APBA/6-PICA	SWV	500–1 × 10^5^	110	31
AuNPs on annealed gold island	DPV	50–3 × 10^4^	5.7	28
Apt/Co-MOF	DPV	80–10^4^	20	This work

**Fig. 5:**
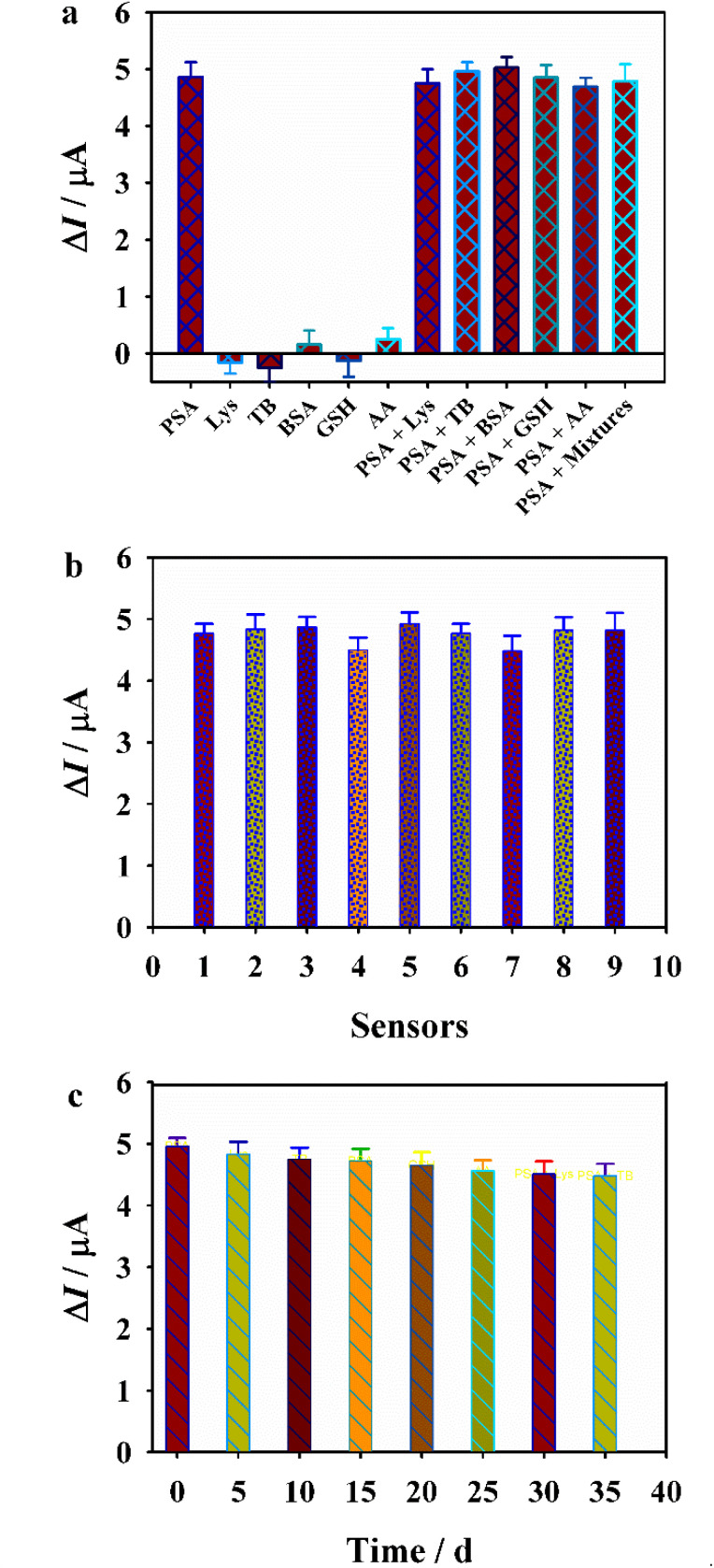
(a) Specificity, (b) reproducibility and (c) stability of the designed Apt/Co-MOF sensor.

To evaluate the selectivity and specificity of the as-developed “off-on” sensing platform, its response was measured toward PSA as well as potential interfering substances, including Lys, TB, BSA, GSH, and AA (Fig. 5a). Results demonstrated that only PSA (0.4 ng mL^−1^) induced a notable decrease in the current signal, while other substances, even at six times the concentration of PSA, produced negligible changes in Δ*I*. Furthermore, electrochemical responses were examined in the presence of PSA mixed with these interferents, and no significant variation was observed in the DAP signal. These findings confirmed the high selectivity and specificity of the “off-on” sensor. Furthermore, to assess reproducibility, nine independently prepared Apt/Co-MOF/GCE sensors were used to measure the DAP current response upon incubation with 0.4 ng mL^−1^ PSA. As depicted in Fig. 5b, the relative standard deviation (RSD) of the Δ*I* values was only 4.37%, indicating excellent reproducibility. The stability of the sensor was also investigated. After storage for 35 days, the sensor retained 90.2% of its initial current response (Fig. 5c), demonstrating satisfactory long-term stability.

The practical applicability of the “off-on” sensor was validated using human serum, which was obtained from the First Affiliated Hospital of Nanjing Medical University, and the initial concentration of PSA could not be detected. Therefore, the standard addition method was introduced to confirm the practical applicability. In detail, different concentrations of PSA were spiked into the serum samples, and the corresponding electrochemical responses were recorded to determine the PSA levels. Meanwhile, the accuracy of the proposed method was validated by comparing its results for spiked samples against those of the ELISA technique. As summarized in Table S1, the recovery rates ranged from 92.7–96.5%, with RSD values between 2.64% and 3.24%, and the results obtained from this proposed method and ELISA were consistent. These findings demonstrate that the developed “off-on” sensor is suitable for detecting PSA in real biological samples. Finally, in order to study the generalizability of the sensing platform, the as-designed “off-on” sensor was used to detect carcinoembryonic antigen (CEA) by just altering the relative Apt. As shown in Fig. S6, the obtained results demonstrated that the decrease in DAP current using Apt/Co-MOF/GCE was smaller than that using Co-MOF/GCE. After the addition of CEA target, the response increased. These findings revealed that this sensing strategy is suitable for detecting other biomarkers by using a different Apt.

## 4. Conclusions

In this work, an innovative “off-on” sensing strategy controlled by Apt was developed based on the OXD-like nanozyme activity of a Co-MOF nanohybrid for the detection of PSA. The prepared Co-MOF nanozyme demonstrated the ability to directly catalyze the oxidation of OPD to generate DAP, eliminating the need for H_2_O_2_, which is typically required in POD-like nanozyme systems. When Apt was immobilized onto the Co-MOF surface to form the Apt/Co-MOF sensing interface, the OXD-like activity was significantly suppressed, leading to a decrease in the DAP peak current (signal “off”). Upon the introduction of PSA, the catalytic activity was restored, and the resulting DAP signal showed a linear increase with PSA concentration, corresponding to the signal “on” state. After the optimization of key experimental conditions, the proposed “off-on” sensor exhibited an excellent PSA detection performance, with an LOD of 20 pg mL^−1^ and a linear range of 80–10000 pg mL^−1^. This simple, OXD-mimicking nanozyme-based “off-on” sensing platform offers a promising new approach for the electrochemical detection of PSA and other biomarkers, demonstrating considerable potential for practical applications.

## Conflicts of interest

There are no conflicts of interest to declare.

## Supplementary Material

RA-016-D6RA01552B-s001

## Data Availability

Data will be made available on request. Supplementary information (SI) is available. See DOI: https://doi.org/10.1039/d6ra01552b.
