# Supplementary material for: Nanoflower-shaped cobalt-metal–organic framework as an oxidase-like nanozyme and 1,2-diaminobenzene as a catalytic substrate for the innovative signal “off-on” aptamer sensing of prostate-specific antigen
Source: RSC Adv. 2026 May 5;16(26):23288–94. doi: 10.1039/d6ra01552b (PMC13140283; doi:10.1039/d6ra01552b)
Supplement: RA-016-D6RA01552B-s001 [file RA-016-D6RA01552B-s001.pdf]

Supplementary Information (SI) for RSC Advances.
This journal is © The Royal Society of Chemistry 2026

Supporting information

Cobalt-metal-organic frameworks nanoflower as oxidase-like nanozyme and 1,2-diaminobenzene as catalytical substrate for innovatively signal "off-on" aptamer sensing of prostate-specific antigen

Chen Ji,[a#] Yi Zhang,[a#] Xingtian Wang,[a] Pingying Xie,[a] Shaoting Wu,[c*] Yanfang Zheng,[b*] Mingqing Huang [b*]

[a] Department of Urology, The Affiliated People's Hospital of Fujian University of Traditional Chinese Medicine, Fuzhou 350004, China

[b] The Affiliated People's Hospital, College of Pharmacy, Fujian University of Traditional Chinese Medicine, Fuzhou 350122, China

[c] Department of Clinical Neurophysiology The Affiliated People's Hospital of Fujian University of Traditional Chinese Medicine, Fuzhou 350004, China

[#] Co-first authors.

[*] Corresponding authors: hmq1115@126.com (M. Huang); 815181526@qq.com (S. Wu); yfzheng@fjtcm.edu.cn (Y. Zheng).

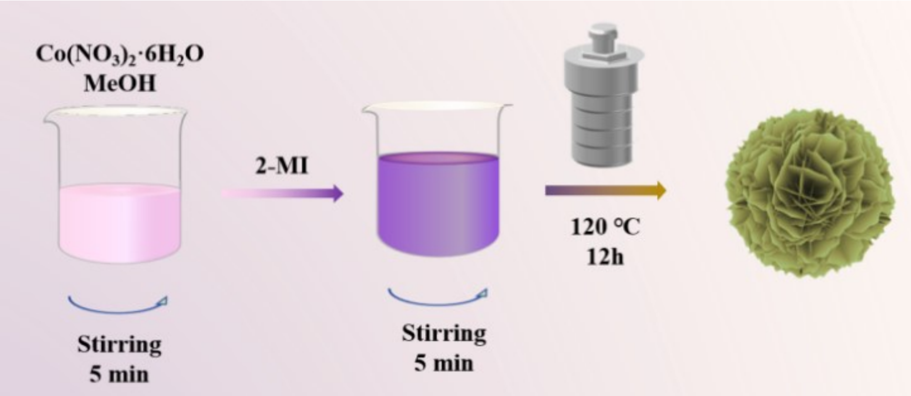

Scheme S1. Schematic illustration for the preparation of Co-MOF nanozyme.

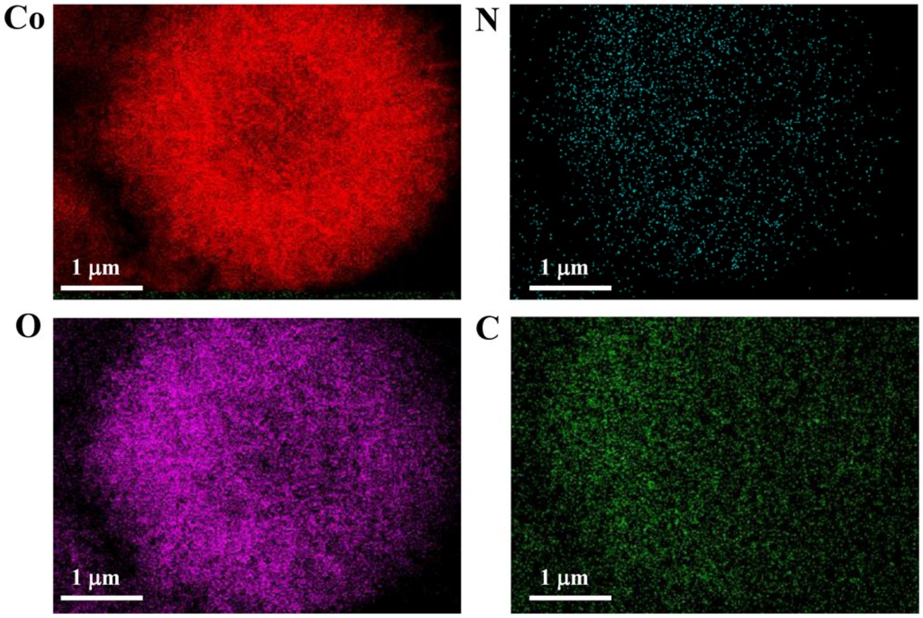

Figure S1. EDS elemental maps of Co-MOFs.

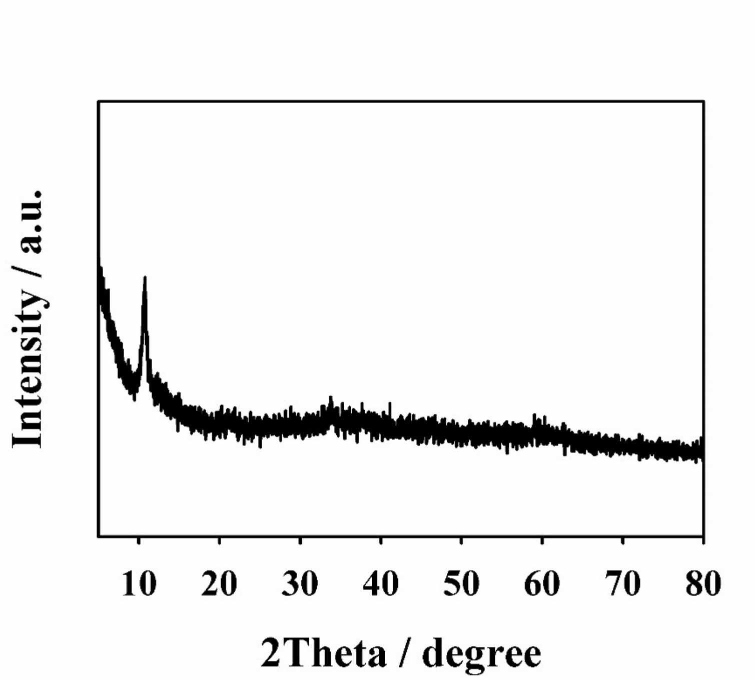

Figure S2. PXRD pattern of Co-MOFs.

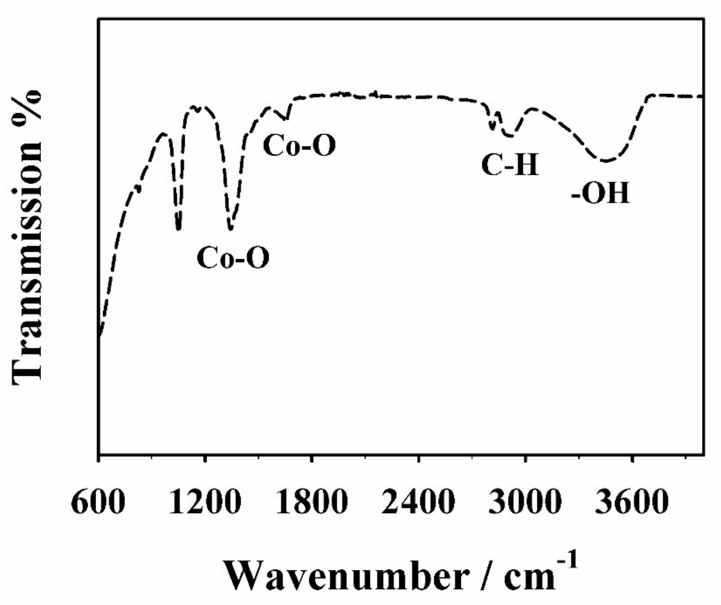

Figure S3. FTIR spectrum of Co-MOFs.

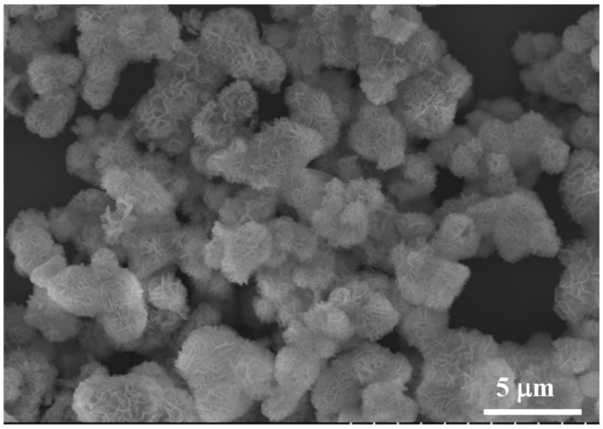

Figure S4. The SEM image of Co-MOFs nanohybrids on the surface of GCE.

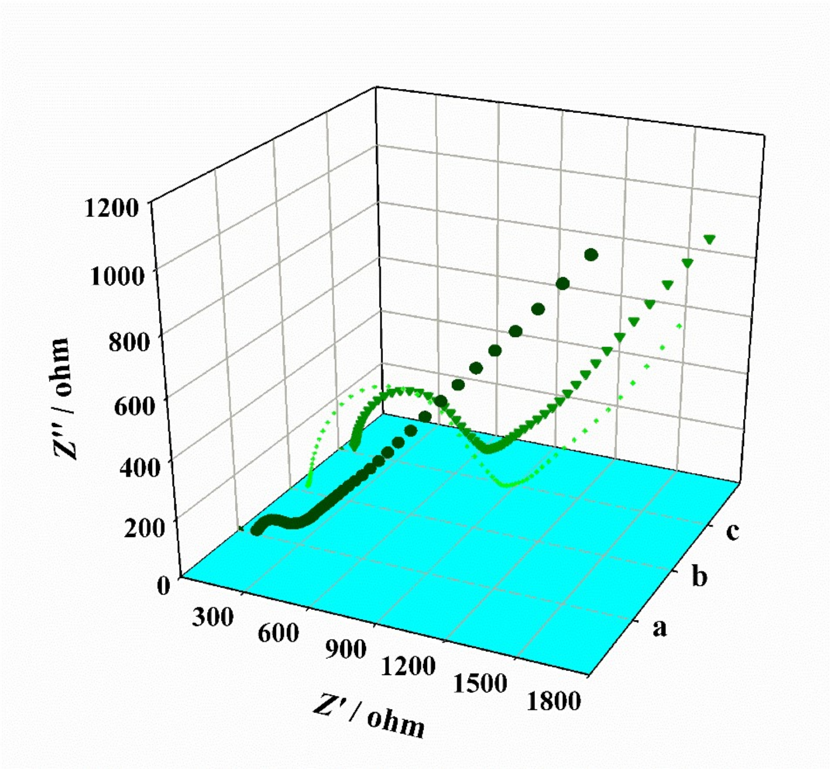

Figure S5 EIS plots of Co-MOFs/GCE (a), Apt/Co-MOFs/GCE before (b) and after (c) incubation with PSA in 0.1 M KCl solution containing 5 mM $[Fe(CN)_6]^{3-/4-}$.

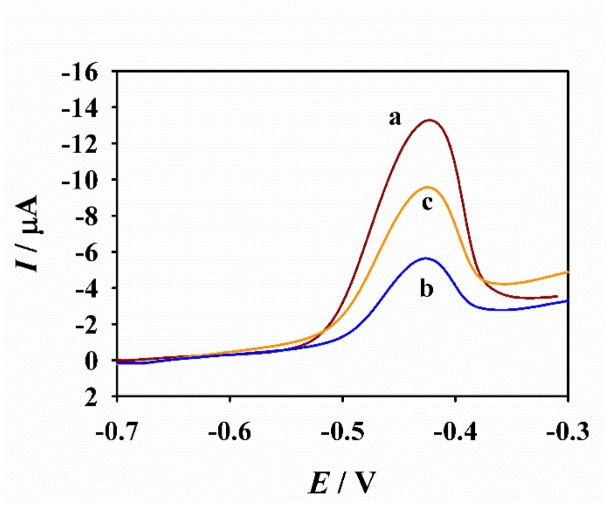

Figure S6. DPV response signals of DAP at Co-MOFs/GCE (a), Apt/Co-MOFs/GCE before (b) and after (c) incubation with CEA.

Table S1. PSA recovery tests in human serum samples.

Sample	Added (pg/mL)	Detected (pg/mL)	Recovery	RSD (%)	ELISA
a	300	286.5	95.5	3.24	292.3
b	500	463.6	92.7	2.85	476.5
c	800	772.4	96.5	2.64	785.6
d	2000	1983.6	99.18	2.57	1990.2
